# cGAS/STING-mediated **γδ** T cell recruitment drives radioresistance: implications for improving radioimmunotherapy outcomes

**DOI:** 10.1172/JCI200465

**Published:** 2025-12-15

**Authors:** Brooke C. Braman, David R. Raleigh

**Affiliations:** 1 Department of Radiation Oncology,; 2 Department of Neurological Surgery, and; 3 Department of Pathology, UCSF, San Francisco, California, USA.

## Abstract

Radiotherapy is a key treatment modality in many malignancies, but radiation-induced immunosuppression can undermine its outcomes and diminish the efficacy of combinatorial strategies, like radioimmunotherapy. In this issue of the JCI, Deng et al. implicate cGAS/STING signaling in the recruitment of γδ T cells that drive downstream radioresistance. Radiation-induced microparticles containing double-stranded tumor DNA led to activation of the cGAS/STING pathway in macrophages, promoting γδ T cell recruitment through CCL20 signaling. In mouse models, γδ T cell–dependent recruitment of myeloid-derived suppressor cells and T cell suppression curbed radiotherapy efficacy and drove antitumor immunity. Ablation of γδ T cells improved the efficacy of radiotherapy alone and radiotherapy combined with immune checkpoint inhibitors in mouse models, supporting further investigation of γδ T cell targeting to improve clinical outcomes with radioimmunotherapy. The findings also add complexity to the function of the cGAS/STING pathway in setting the balance between antitumor immunity and immunosuppression.

## Introduction

Cancer immunotherapy dates back to at least the late 19^th^ century, when injection of inactivated bacterial cultures yielded some instances of durable tumor control in sarcoma (1). Over the past 20 years, immunotherapy, particularly immune checkpoint inhibitors (ICIs), has revolutionized clinical oncology. Groundbreaking work that untangled mechanisms of immune tolerance and evasion by cancer cells laid the foundation for development of ICIs. In 2011, the FDA approved the first ICI, ipilimumab, an anti-CTLA4 monoclonal antibody (2), after it was shown to nearly double overall survival for patients with previously treated metastatic melanoma (3). Ipilimumab’s approval was swiftly followed by the evaluation and approval of the anti-PD1 monoclonal antibody, pembrolizumab (2). Pembrolizumab was found to outperform ipilimumab in advanced melanoma (4) and now has the most FDA-approved indications of any cancer immunotherapy (5). First investigated and implemented as monotherapies, these agents have subsequently been evaluated in combination with other oncologic treatments, such as radiotherapy, with disappointingly mixed results.

## Radioimmunotherapy yields mixed results in clinical trials

Radiotherapy is used as a treatment in approximately half of all patients with cancer. The ability of radiotherapy to potentiate antitumor immunity via stimulation of neoantigen presentation and cytokine release has inspired numerous clinical trials investigating its combination with ICIs (6, 7). Some trials have demonstrated success, like PACIFIC and KEYNOTE-A18, in which ICIs added to chemoradiotherapy in locally advanced non-small lung cancer (NSCLC) (8) and high-risk, locally advanced cervical cancer, respectively, led to improvements in progression-free survival and overall survival (9). However, no benefit has been demonstrated in many other clinical trials, including in varied advanced and metastatic solid tumor settings (10, 11), in locally advanced head and neck squamous cell carcinoma (12), and in glioblastoma (13, 14), the most common malignant brain tumor in adults. Radiotherapy-induced death of radiosensitive lymphocytes and limited immune cell infiltration or exhausted T cell phenotypes in some tumors have been implicated in the failure of these trials (6, 15). Thus, radiotherapy’s ability to promote immunosuppression in addition to immune activation has been increasingly recognized as a challenge to optimizing outcomes of both radiotherapy-only and combined approaches.

The robust investigation by Deng et al. (16) in this issue of the JCI provides a mechanistic explanation for radiotherapy-induced immunosuppression that drives radioresistance, paving the way for a potential strategy to improve the efficacy of radioimmunotherapy.

## γδ T cells mediate immunosuppressive responses to radiotherapy

**Figure 1:**
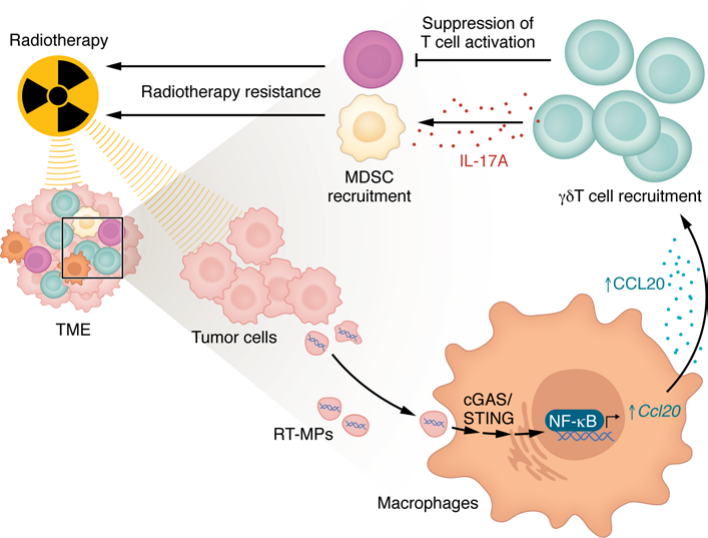
γδ T cell recruitment mediates immune suppression that drives radioresistance. Through a series of robust experiments, Deng et al. (16) showed that radiation-induced, tumor-derived microparticles (RT-MPs) stimulate CCL20 production in macrophages via the cGAS/STING pathway. CCL20 then recruits γδ T cells, which produce immunosuppression and radioresistance through suppression of T cell activation as well as IL-17A–mediated recruitment of myeloid-derived suppressor cells (MDSCs). The findings support further investigation of strategies targeting cGAS/STING signaling as well as γδ T cells to improve response to cancer therapy.

In a series of carefully constructed in vitro experiments and murine models leveraging multiple sequencing techniques, gene ontology enrichment analysis, cytokine characterization, and chemotaxis assays, Deng et al. identified γδ T cells as potent mediators of radioresistance in multiple models of cancer across varied radiation regimens (summarized in Figure 1). In in vitro models, they demonstrated that radiation-induced tumor cell–derived microparticles (RT-MPs) containing double-stranded DNA (dsDNA) upregulated the cGAS/STING/NF-κB pathway in macrophages to stimulate CCL20 production. CCL20 production led to recruitment of γδ T cells, a unique population of innate-like T cells that respond to nonclassical antigens, including tumor neoantigens. Degradation of dsDNA in RT-MPs, CCL20 blockage, STING inhibition, and macrophage depletion inhibited γδ T cell recruitment. In a murine model of lung cancer, they showed that γδ T cells secreted IL-17A to recruit myeloid-derived suppressor cells (MDSCs) and suppress T cell activity. Both of these downstream effects of IL-17A secretion hindered antitumoral immunity and limited response to radiotherapy in mice. Further, ablation of γδ T cells or IL-17A blockade or MDSC depletion combined with intact γδ T cell populations yielded improved tumor regression and survival after radiotherapy in murine models. Finally, Deng and colleagues showed that the addition of radiation to anti-PD1 therapy improved therapeutic efficacy in mice with ablated γδ T cells compared with anti-PD1 monotherapy or when γδ T cells were present, suggesting a potential strategy to overcome radioresistance and potentiate radioimmunotherapy efficacy in patients. Indeed, Deng et al. connected their preclinical findings to human data by showing upregulation of γδ T cell–specific genes in irradiated tumor tissue from patients with pancreatic ductal adenocarcinoma and upregulation of both γδ T cells and IL-17A in serum from patients with NSCLC after radiotherapy.

## Duality in the cGAS/STING pathway: radioresistance versus radiosensitivity

The study’s description of cGAS/STING’s role in immune suppression resulting in radioresistance (16) adds to the complexity of this pathway, which is also known to promote antitumoral immunity. In addition to recruiting immunosuppressive γδ T cells, cGAS/STING activation has been shown to enhance tumor cell killing by upregulating multiple proinflammatory cytokines, principally IFN, and upregulating costimulatory molecules required for antigen presentation. Preclinical work has helped define cGAS/STING’s role in increased antitumoral activity and response to radiotherapy. For example, tumor-bearing STING-deficient mice have been shown to exhibit radioresistance, suggesting that STING also has a role in radiosensitivity (17). In STING-intact mice, cGAS/STING activation in dendritic cells after direct sensing of irradiated tumor cells promotes cytotoxic T cell–driven antitumor responses through type 1 IFN signaling, and increased STING activation through exogenous cGAMP improves radiotherapy efficacy (17). Other work has demonstrated that the addition of a STING agonist to low-dose radiotherapy and anti-PD1 therapy yields superior tumor regression at primary and distant sites and improved survival in murine models (18). The combined activity of radiation and STING agonist–derived IFN upregulation mediates these effects through improved immune infiltration of CD8^+^ T cells, dendritic cells, NK cells, and M1 macrophages (18). Radiation-induced cGAS/STING upregulation thus exhibits a duality of antitumoral immune activation and suppression that influences both radioresistance and radiosensitivity. Competing effects like these help explain the mixed outcomes seen across a spectrum of clinical implementations of radioimmunotherapy. Whether the balance of these opposing forces is tumor intrinsic or is dependent on radiation regimen requires further clarification. Elucidation of these enduring questions may better inform the design of future clinical trials of combined radioimmunotherapy.

## Optimizing antitumor immunity in clinical contexts

More broadly, the immune stimulatory properties of cGAS/STING activation have given rise to interest in STING agonists as anticancer therapies, especially in immunologically “cold” tumors that are resistant to immunotherapy approaches, like glioblastoma. Multiple orthotopic murine models of glioblastoma mimic the human phenotype with limited baseline tumor immune infiltration; use of STING agonists in these cold models promoted influx and activation of antitumoral immune effector cells while reducing immune exhaustion and increasing survival, including in mice with humanized immune systems (19, 20). Subsequent addition of anti-PD1 therapy to a STING agonist further improved survival in an immunologically “hot” murine model of glioblastoma, but not in an immunologically cold model, suggesting that additional immune modulation may be required to promote maximal antitumoral immune response in this setting (19). Nonetheless, promising preclinical data like these have inspired the evaluation of STING agonists alone and in combination with ICIs in early phase clinical trials across multiple disease sites (21). Given the ubiquity of radiotherapy in cancer care, the complex interactions of these therapies with a tumor microenvironment that has been remodeled by radiation should be considered, lest STING activation inadvertently contribute to immune suppression and radioresistance, as shown by Deng et al. (16).

Additional strategies to further prime the tumor microenvironment will likely be needed to fully realize the potential of immunotherapy, especially when used as multimodal therapy. For example, IL-6 has been reported to contribute to immunosuppression in glioblastoma through PD-L1 upregulation in myeloid cells, and higher IL-6 expression has been correlated with worse survival in glioblastoma (22). Emerging work using spatial protein profiling of human glioblastoma tissue samples paired before and after exposure to ICI has identified enrichment in IL-6 and its downstream effectors in ICI nonresponders (23). Combined IL-6 and PD-1 blockade remodels the tumor microenvironment towards an antitumoral phenotype, albeit transiently, and improves survival in an allograft murine model of glioblastoma whose tumor microenvironment resembles that of the human disease (23). The addition of this dual blockade also improves survival in murine models when added to high-dose rate radiation compared with radiation monotherapy, suggesting this as a potential strategy to enhance radiosensitivity through augmented immune activation (23). This promising strategy is the basis of the ongoing clinical trial NRG-BN010 (NCT04729959), which is investigating the combination of tocilizumab, atezolizumab, and fractionated stereotactic radiotherapy in recurrent glioblastoma (24). While combined ICIs and radiotherapy have not been universally successful thus far, continued investigation into the overlapping and opposing pathways driving treatment resistance is likely to inform their future use.

## Funding support

This work is subject to the NIH Public Access Policy. This work is the result of NIH funding, in whole or in part, and is subject to the NIH Public Access Policy. Through acceptance of this federal funding, the NIH has been given a right to make the work publicly available in PubMed Central.

- NIH grants U54 CA302452, P01 CA118816, UH3 CA298961, R21 NS141756, R01 CA262311, R01 CA251221, and U19 CA264338.

## Version 1 12/15/2025

Electronic publication

## References

[1] Decker WK (2017). Cancer Immunotherapy: Historical Perspective of a Clinical Revolution and Emerging Preclinical Animal Models. Front Immunol.

[2] Korman AJ (2022). The foundations of immune checkpoint blockade and the ipilimumab approval decennial. Nat Rev Drug Discov.

[3] Hodi FS (2010). Improved survival with ipilimumab in patients with metastatic melanoma. N Engl J Med.

[4] Robert C (2015). Pembrolizumab versus Ipilimumab in Advanced Melanoma. N Engl J Med.

[5] https://www.accessdata.fda.gov/drugsatfda_docs/label/2020/125514s066lbl.pdf.

[6] Galluzzi L (2023). Emerging evidence for adapting radiotherapy to immunotherapy. Nat Rev Clin Oncol.

[7] Spiotto M (2016). The intersection of radiotherapy and immunotherapy: mechanisms and clinical implications. Sci Immunol.

[8] Spigel DR (2022). Five-Year Survival Outcomes From the PACIFIC Trial: Durvalumab After Chemoradiotherapy in Stage III Non-Small-Cell Lung Cancer. J Clin Oncol.

[9] Duska LR, Xiang Y, Hasegawa K (2025). Pembrolizumab with chemoradiotherapy in patients with high-risk locally advanced cervical cancer: Final analysis results of the phase 3, randomized, double-blind ENGOT-cx11/GOG-3047/KEYNOTE-A18 study. JCO.

[10] Theelen WSME (2019). Effect of Pembrolizumab After Stereotactic Body Radiotherapy vs Pembrolizumab Alone on Tumor Response in Patients With Advanced Non-Small Cell Lung Cancer: Results of the PEMBRO-RT Phase 2 Randomized Clinical Trial. JAMA Oncol.

[11] Spaas M (2023). Checkpoint Inhibitors in Combination With Stereotactic Body Radiotherapy in Patients With Advanced Solid Tumors: The CHEERS Phase 2 Randomized Clinical Trial. JAMA Oncol.

[12] Lee NY (2021). Avelumab plus standard-of-care chemoradiotherapy versus chemoradiotherapy alone in patients with locally advanced squamous cell carcinoma of the head and neck: a randomised, double-blind, placebo-controlled, multicentre, phase 3 trial. Lancet Oncol.

[13] Omuro A (2023). Radiotherapy combined with nivolumab or temozolomide for newly diagnosed glioblastoma with unmethylated MGMT promoter: An international randomized phase III trial. Neuro Oncol.

[14] Lim M (2022). Phase III trial of chemoradiotherapy with temozolomide plus nivolumab or placebo for newly diagnosed glioblastoma with methylated MGMT promoter. Neuro Oncol.

[15] Zhang J (2022). Turning cold tumors hot: from molecular mechanisms to clinical applications. Trends Immunol.

[16] Deng Y (2025). IL-17-producing γδ T cells in the tumor microenvironment promote radioresistance in mice. J Clin Invest.

[17] Deng L (2014). STING-Dependent Cytosolic DNA Sensing Promotes Radiation-Induced Type I Interferon-Dependent Antitumor Immunity in Immunogenic Tumors. Immunity.

[18] Zheng Y (2025). Stimulator of Interferon Genes Agonist Synergistically Amplifies Programmed Cell Death Protein-1 Blockade and Radiation-Induced Systemic Antitumor Responses via Tumor Microenvironment Enrichment. Int J Radiat Oncol Biol Phys.

[19] Najem H (2024). STING agonist 8803 reprograms the immune microenvironment and increases survival in preclinical models of glioblastoma. J Clin Invest.

[20] Berger G (2022). STING activation promotes robust immune response and NK cell-mediated tumor regression in glioblastoma models. Proc Natl Acad Sci U S A.

[21] Wang B (2024). Clinical applications of STING agonists in cancer immunotherapy: current progress and future prospects. Front Immunol.

[22] Lamano JB (2019). Glioblastoma-Derived IL6 Induces Immunosuppressive Peripheral Myeloid Cell PD-L1 and Promotes Tumor Growth. Clin Cancer Res.

[23] https://www.doi:10.1101/2025.03.12.642800.

[24] Bagley S, Polley MY, Kotecha R, Bren S (2022). CTIM-21. NRG-BN010: A Safety Run-In and Phase II Study Evaluating the Combination of Tocilizumab, Atezolizumab, and Fractionated Stereotactic Radiotherapy in Recurrent Glioblastoma. Neuro-Oncol.

